# Universal School-Based Depression Prevention ‘Op Volle Kracht’: a Longitudinal Cluster Randomized Controlled Trial

**DOI:** 10.1007/s10802-015-0080-1

**Published:** 2015-09-25

**Authors:** Yuli R. Tak, Anna Lichtwarck-Aschoff, Jane E. Gillham, Rinka M. P. Van Zundert, Rutger C. M. E. Engels

**Affiliations:** Behavioural Science Institute, Radboud University Nijmegen, P.O. Box 9104, 6500 HE Nijmegen, The Netherlands; Psychology Department, Swarthmore College, 500 College Avenue, Swarthmore, PA 19081 USA; The Penn Resiliency Project, Positive Psychology Center, University of Pennsylvania, Philadelphia, PA USA; Trimbos-Institute, P.O. Box 725, 3500 AS Utrecht, The Netherlands; Leer & Veerkracht, Havenstraat 15b, 5211 WC’s, Hertogenbosch, The Netherlands

**Keywords:** Depressive symptoms, Depression, Universal prevention, Adolescence

## Abstract

The longitudinal effectiveness of a universal, adolescent school-based depression prevention program Op Volle Kracht (OVK) was evaluated by means of a cluster randomized controlled trial with intervention and control condition (school as usual). OVK was based on the Penn Resiliency Program (PRP) (Gillham et al. *Psychological Science, 6*, 343–351, 1995). Depressive symptoms were assessed with the Child Depression Inventory (Kovacs 2001). In total, 1341 adolescents participated, *M*age = 13.91, *SD* = 0.55, 47.3 % girls, 83.1 % Dutch ethnicity; intervention group *n* = 655, four schools; control group *n* = 735, five schools. Intent-to-treat analyses revealed that OVK did not prevent depressive symptoms, *β* = −0.01, *SE* = 0.05, *p* = .829, Cohen’s *d* = 0.02, and the prevalence of an elevated level of depressive symptoms was not different between groups at 1 year follow-up, *OR* = 1.00, 95 % CI = 0.60–1.65, *p* = .992, NNT = 188. Latent Growth Curve Modeling over the 2 year follow-up period showed that OVK did not predict differences in depressive symptoms immediately following intervention, intercept: *β* = 0.02, *p* = .642, or changes in depressive symptoms, slope: *β* = −0.01, *p* = .919. No moderation by gender or baseline depressive symptoms was found. To conclude, OVK was not effective in preventing depressive symptoms across the 2 year follow-up. The implications of these findings are discussed.

Depressive disorder is one of the most prevalent mental disorders in adolescence (Merikangas et al. 2010) and is associated with social impairments (Birmaher et al. 1996; Carr 2006), suicide (Bridge et al. 2006), and high treatment costs (Meijer et al. 2006). Even experiencing elevated levels of depressive symptoms put adolescents at risk for depressive disorder (Seeley et al. 2009), and diminished academic, peer and family functioning (Gotlib et al. 1995; Jaycox et al. 2009). At age 11, 10 % of children experience elevated levels of depressive symptoms, and by age 15 this has increased to 24.5 % (Saluja et al. 2004), with girls outnumbering boys 2:1 (Hankin et al. 2007; Twenge and Nolen-Hoeksema 2002). Therefore, preventing the onset and increased prevalence of depressive symptoms is of great importance.

Depression prevention can be targeted at individuals with elevated symptoms (indicated prevention), or other risk factors for depression (selective prevention), or it can be administered to all individuals in a setting regardless of their individual level of symptoms or risk status (universal prevention). Universal depression prevention programs can effectively prevent depressive symptoms in the short term (Merry et al. 2012). Compared to universal approaches, targeted approaches have been found to more effectively prevent depressive symptoms (Stice et al. 2009). As a result, some scholars currently question the value of universal depression prevention (Merry 2013; Spence et al. 2003, 2005). While some universal prevention programs were effective in preventing depressive symptoms at post assessment, these effects did not maintain over time (Challen et al. 2014; Spence et al. 2003; Spence et al. 2005). Additionally, recent universal prevention trials reported no benefits. For example: the Beyond Blue program in Australia (Sawyer et al. 2010), The I Think, Feel and Act program in Chile (Araya et al. 2013), and the RAP program in the UK (Stallard et al. 2012). However, it should be noted that Stallard et al. (2012) reported program effectiveness for adolescents displaying high depressive symptom levels and not for all participating adolescents, and Araya et al. (2013) included adolescents from deprived socioeconomic areas only.

In contrast to these results, some universal prevention programs showed long-term benefits from 4 months up to 18 months follow-up (Lock and Barrett 2003; Merry et al. 2004; Pössel et al. 2004; Pössel et al. 2013; Pössel et al. 2008; Shochet et al. 2001). In addition to these results, universal depression prevention programs have several advantages compared to targeted approaches: they can be embedded within school curriculum so that all adolescents might improve their coping skills and well-being (Tugade et al. 2004), they avoid the difficulties of identifying adolescents at risk for depression, and they minimize the risk of stigmatizing at-risk adolescents (Barrett et al. 2006). Given these advantages and the mixed results of universal depression prevention, we should continue to develop and investigate universal depression prevention programs.

The annual prevalence of depressive disorder among Dutch adolescents is high – 3.8 % (37,400) of those between age 13 and 17 suffer from depression (Meijer et al. 2006). Still, theory-based and validated universal adolescent depression prevention in the Netherlands is lacking (Schrijvers and Schoemaker 2008). Therefore, the current study evaluated an adapted version of the previously successful Penn Resiliency Program (PRP) (Brunwasser et al. 2009) in the Netherlands. PRP was therefore adapted to the Dutch culture and named “Op Volle Kracht” (OVK) which translates to: At Full Force (Tak et al. 2012). When used as an indicated prevention program, OVK was found to reduce depressive symptoms at 6 months follow-up in adolescent girls with elevated levels of depressive symptoms (Wijnhoven et al. 2014). However, when OVK was implemented as a selective prevention program, it did not prevent depressive symptoms in Dutch youth (Kindt et al. 2014).

OVK and PRP are based on several psychological theories and principles, namely Cognitive Behavioral Therapy (CBT) (Beck 1976), the ABC model (Ellis 1962), and the hopelessness theory of depression (Hankin et al. 2001). CBT-based intervention and prevention programs have been found to effectively reduce and prevent depressive symptoms in adolescents (for a review see Butler et al. 2006). In OVK therefore, adolescents learn that their thoughts influence their feelings and their behavior. They learn to identify their negative cognitions and to replace them with realistic ones. Moreover, since adaptive coping is associated with lower levels of depressive symptoms (Calvete et al. 2011), OVK aims to improve adolescents’ coping skills, problem solving, and decision making (Jaycox et al. 1994; Tak et al. 2012).

Thus, OVK is closely modeled off of PRP. However, while several studies provide evidence for PRP’s effectiveness, PRP has never been tested with a randomized controlled design in a true universal form. Several studies evaluating PRP were designed as universal tests, but in these studies less than 25 % of the adolescents that were initially invited to participate were eventually included (Chaplin et al. 2006; Gillham et al. 2007). These trials cannot be considered “universal”, since they included only a sub-set of the sample that was invited to participate. Even studies that strictly adhered to a universal approach suffered from methodological shortcomings. In Challen et al. (2014), the PRP program was tested in a universal sample with a controlled design, but lacked random assignment. In the trial by Silverstone et al. (2015), only the first eight lessons of OVK were administered. The current study was therefore the first to employ a randomized controlled design to examine the longitudinal effectiveness of the complete OVK program as a universal, school-based depression prevention program. Our main outcome assessment is the 1 year follow-up assessment, since mastering and applying the program’s skills in daily life takes time (Brunwasser et al. 2009). The effectiveness of OVK in both reducing the level of depressive symptoms and the number of adolescents experiencing elevated levels of depressive symptoms will be analyzed.

Previous research has highlighted the importance of investigating moderators of intervention effects in order to understand for whom prevention programs are most beneficial (Stice et al. 2009). In the current study, we investigated whether gender and whether high levels of depressive symptoms at baseline would moderate intervention effects. In previous PRP studies, conflicting results have been found regarding gender differences: one study revealed better results for boys (Reivich 1996), whereas another study revealed better results for girls (Gillham et al. 2006). Given these opposing results, no specific hypotheses were formulated regarding gender as moderator. Since prevention programs among adolescents at risk or with elevated symptoms at baseline often report larger effect sizes (Cardemil et al. 2002; Merry et al. 2012; Stice et al. 2009), we hypothesized that adolescents reporting elevated levels of depressive symptoms at baseline would show a larger intervention effect than those with few or no symptoms.

## Method

### Participants & Procedure

Schools providing secondary education in the southern and middle part of the Netherlands were invited to participate in the study. The Netherlands offers several types of secondary education: VMBO refers to pre-vocational secondary education which prepares pupils to be admitted to a vocational or technical institute; HAVO refers to higher general secondary education which prepares students for select Bachelor programs; VWO refers to pre-university education which enables students to apply to all universities and Bachelor programs (Dutch Ministry of Education and C. a. S 2014). All adolescents in the eighth grade from participating schools were eligible to participate. Adolescents were included through passive consent, but were free to withdraw from the study and participation in the OVK lessons at any point if their parents or they themselves wanted to discontinue participation. As the intervention was incorporated into the school curriculum and administered during school hours, school principals had to give active consent for their school’s participation. Parents and adolescents were informed about the research by mail. In schools allocated to the intervention condition, a presentation was delivered to inform parents and teachers about the OVK program and study aims.

Assessments were conducted by administering questionnaires to adolescents during school hours. Depending on the schools’ preferences, the questionnaire was provided either on paper or digitally. At all assessment points, questionnaires were administered by a research assistant or a teacher. At the follow-up assessments, adolescents not in attendance were asked to complete the questionnaire at home. To maintain a high retention rate, these adolescents were contacted via mail, email, and subsequently via telephone to complete the questionnaire. Adolescents received a gift voucher of €7.50 (approximately $8.50) for every follow-up questionnaire completed outside school hours. Also to maintain a high retention rate at 1 year follow-up, five additional gift vouchers of €20 (approximately $23) were randomly awarded to adolescents who completed the assessment outside of school hours. At the 2 year follow-up assessment, all participating adolescents received a gift voucher of €7.50.

Baseline assessment was conducted in January 2011 and the OVK program was provided from February to June, 2011. Five follow-up assessments were conducted: at post intervention, and every 6 months up to 2 years follow-up. The 1 year follow-up assessment was administered between June and August, 2012. Following the 6 month follow-up, a 2 h booster session of was provided to all schools in the intervention condition in February or March, 2012. In this same period, the teachers from schools in the control condition received a training in OVK as a reward for their school’s participation. These teachers were instructed not to deliver OVK to adolescents still participating in the study. The research protocol and trial design as registered by the Dutch Trial Registration (NTR2879) and were approved by the ethics committee of the Faculty of Social Sciences at the Radboud University Nijmegen (number 16122010).

### Trial Design & Randomization

A two-arm parallel cluster randomized controlled design was used to test the longitudinal effectiveness of OVK in preventing depressive symptoms in adolescents. Schools were approached for study participation, but the individual adolescents were the unit of analysis. To minimize contamination between those in the OVK intervention and those in the control condition (lessons as usual), schools were randomly assigned to condition, with the intent to randomize with an allocation ratio of 1:1. Randomization of schools to condition was conducted by an independent statistician from Utrecht University after active consent from all school principals was received. The schools that participated in this study differed in the types of education they provided and the proportion of students in each educational track. The schools could offer a combination of pre-vocational secondary education (PVSE), higher general secondary education (HGSE), or pre-university education (PUE). To therefore ensure a more even distribution of the different types of education across conditions, the randomization was stratified by type of education. Adolescents, principals and teachers were not blinded to research condition. In the intervention condition, classes were split in two to create smaller groups, resulting in 10–16 students per group. In some schools, the teachers determined the group composition and aimed to distribute students who generally showed more disruptive behavior evenly across the two groups. OVK was provided during mentor lessons, in which the teacher responsible for this class facilitates discussions about school organization, homework, school related problems, and organizing a classroom event. The control group received the mentor lesson as usual. For adolescents in the intervention condition, matters related to school organization, homework, school related problems and organizing a classroom event, were discussed between lessons or in other lessons. This might have resulted in slightly less discussion time for these matters.

### Intervention

The prevention program Op Volle Kracht (OVK) is based on PRP (Gillham et al. 1995), but adaptations were made to better fit our design and sample (for a detailed program description see Tak et al. 2012). First, the examples provided in the PRP booklet were made more relevant for Dutch youth, and the layout of the booklet was made more modern and colorful. Adaptations were also made to PRP’s scheduling and content to best implement OVK within the school curriculum. Whereas PRP consists of 12 lessons lasting 90 min each, OVK consisted of 16 lessons lasting 50 min each, and a 2 h booster session delivered at 12 months follow-up. Program duration of OVK is comparable to PRP and other prevention programs (Araya et al. 2013; Pössel et al. 2013; Stallard et al. 2012). Whereas in PRP the relation between a situation, thoughts, feelings and behavior is explained in one session, in OVK these concepts were discussed in separate sessions. Subsequently, in OVK one session was added which combined these four concepts. Additionally, a session on social skills was added. Finally, we somewhat condensed PRP because we expected to need less discussion time in a universal context and because shorter programs have produced better results (Stice et al. 2009).

The first eight lessons of OVK covered the CBT principles and the latter eight lessons were used to practice coping, decision making, social skills, and problem solving skills. All adolescents received a workbook in which all the assignments were presented. Under the supervision of a group trainer, adolescents practiced the skills in a variety of ways: role-playing, holding discussions, or completing pen and paper assignments. Each lesson included homework to facilitate internalization and transfer of the skills. At 8 months follow-up, a 2 h booster session was provided to the students in the intervention condition during school hours. This session began with an assembly which included a hip-hop performance by professional artists who addressed the core principles of OVK. Adolescents subsequently followed a rap workshop with their classmates in which they wrote their own poems about what they had learned from OVK.

OVK was delivered by 10 psychologists (one psychologist per group of students) with varying degrees of experience in Cognitive Behavioral Therapy and teaching; one psychologist was the principal author of this paper. All psychologists (from now on referred to as group trainers) completed a 5 day training in CBT principles and the OVK program. The training was led by two experienced psychologists who had been trained by PRP team members. Most group trainers delivered OVK to five groups, *M* = 5.67, *SD* = 1.40.

To ensure program fidelity, the group trainers and the research team remained in close, frequent contact. Additionally, two meetings were held which included the OVK developers, the research team, and the group trainers. Program fidelity was measured through a self-report questionnaire which was completed by group trainers after each lesson. Group trainers had to indicate for each lesson whether they had discussed each exercise, whether they had discussed the homework, whether they had covered the most important messages, and whether they had assigned homework for the next lesson.

### Measures

#### Depressive Symptoms

Depressive symptoms were measured with the Dutch translation of the Children’s Depression Inventory (CDI) (Kovacs 1985; Timbremont et al. 2008), which has shown to be a reliable and valid measure of depressive symptoms (Evers et al. 2009–2011). The CDI is a 27-item questionnaire where each item consists of three statements; respondents must choose which statement is most consistent with their feelings over the past 2 weeks. For example: ‘I am sad sometimes’ (0), ‘I am often sad’ (1), and ‘I am sad all the time’ (2). Total scores ranged from 0 to 54. Cronbach’s alpha ranged from 0.84 to 0.91 at baseline and follow-ups, indicating very good reliability. Due to ethical considerations, the item concerning suicidal thoughts and ideation was omitted from the questionnaire after the baseline assessment. At baseline and follow-ups mean depression scores and alpha levels were calculated without this item. To enable comparisons between the results of the current paper and past studies, we corrected sum depression scores for the missing item by multiplying mean depression score times 27. Elevated levels of depressive symptoms were determined by dichotomizing depressive symptoms sum scores and were present (1) if CDI sum > = 13, and not present (0) if CDI sum < 13 (Timbremont et al. 2008).

#### OVK-Specific Variables

Attendance to OVK lessons was tracked for all participants. Additionally, adolescents were asked to complete four questions about their experience of the OVK program. Each question was rated on a 4-point Likert-scale 1 (*not true at all*) to 4 (*completely true*), for example: ‘The group atmosphere was characterized as safe’. Also, group trainers completed five questions about the OVK program on a 5-point Likert-scale 1 (*not true at all*) to 5 (*completely true*), for example: ‘Adolescents are motivated for the OVK lessons’.

### Analyses

This study’s primary interest was to test whether OVK was effective at the 1 year follow-up. This study’s primary outcomes are therefore the level of depressive symptoms (continuous) and the presence of an elevated level of depressive symptoms (dichotomous) at 1 year follow-up. Of secondary interest was the effect of OVK across the entire 2 year follow-up period.

A power calculation was performed to enable detection of a low to medium effect (Cohen’s *d* = 0.20) at 1 year follow-up on a dichotomous outcome (CDI = > 13 yes/no), taking into account the clustering of participants in schools, attrition of 20 % over the 2 year follow-up period, and loss of power due to multiple imputation. This resulted in a required sample sizes of at least 662 per condition (alpha < 0.05, power = 0.80).

After randomization, differences between control and intervention condition on gender, age, education, and depressive symptoms at baseline were assessed by means of logistic regression analyses. Two dummy variables were created for education: Lower education represented pre-vocational secondary education (PVSE) = 1, versus higher general secondary education (HGSE) = 0, and pre-university education (PUE) = 0. Middle education represented HGSE = 1, versus PVSE = 0, and PUE = 0. PUE was the reference group. Attrition over the course of the study was analyzed by means of logistic regression analysis in which study drop-out (yes = denied further participation; no = stayed in the study) was the outcome variable and gender, age, education, and depressive symptoms at baseline were predictors.

First, to compare conditions at every follow-up assessment on the continuous and dichotomous outcomes, univariate tests were performed by using *t*-tests and Chi-square tests respectively. Second, the primary intervention effect for the continuous and dichotomous outcome of depressive symptoms at 1 year follow-up was analyzed in Mplus 6.1 (Muthén and Muthén 1998–2010) by means of multiple regression analysis, in which randomization differences and the clustering of participants in schools were accounted for. The Intraclass correlation (ICC) at 1 year follow-up was 0.022, and across all assessments (baseline up to 2 years follow-up), the ICC was 0.036, range: 0.018–0.065. This means that 2.2 and 3.6 % of the variance in depressive symptoms could be explained by school related aspects respectively.

For the continuous outcome only, baseline depressive symptoms were centered. The analyses were conducted according to the completers only (CO) framework (*N* = 1158), and subsequently to the intent-to-treat (ITT) framework (*N* = 1341). Completers only are the adolescents that had completed baseline and the 1 year follow-up assessment. For the ITT analyses only, missing data were imputed 20 times by multiple imputation for the control and intervention group separately as advised by Graham (2009). Imputation was done by means of the predicting mean matching method in SPSS 19. To accurately predict the standard errors, a sufficient number of auxiliary variables were included in the imputation model and the model was imputed several times (Graham 2009). Auxiliary variables are variables that correlated *r* = 0.50–0.60 with depressive symptoms, and were used as predictors of depressive symptoms in the imputation model.

To analyze the effectiveness of OVK on the continuous outcome (the level of depressive symptoms) across the 2 year follow-up period, Latent Growth Curve Modeling was conducted in Mplus 6.1 (Muthén and Muthén 1998–2010) in the ITT framework with imputed data. Three different models were tested. In model one, single growth curves were assessed, in which the intercept was set at post intervention and the slope included all other follow-up assessments (6, 12, 18 and 24 months follow-up). Linear slopes (LS) and Quadratic slopes (QS) were tested. The quadratic slope was only included when the mean and the variance of the slope was significant. In all models, the residual variances of depressive symptoms were free to vary and the intercepts of depressive symptoms were set equal to 0. In model two, baseline depressive symptoms (centered), control variables (age, ethnicity, education), gender, and intervention condition were included to assess their effect on the intercept and slope. In model three, the interactions of baseline depressive symptoms x condition and gender x condition were included. Model fit was considered appropriate when RMSEA was smaller than 0.05, and CFI was larger than 0.90. Since Chi-square results are less reliable with large sample sizes, these are not reported (Raykov and Marcoulides 2006).

## Results

**Fig. 1:**
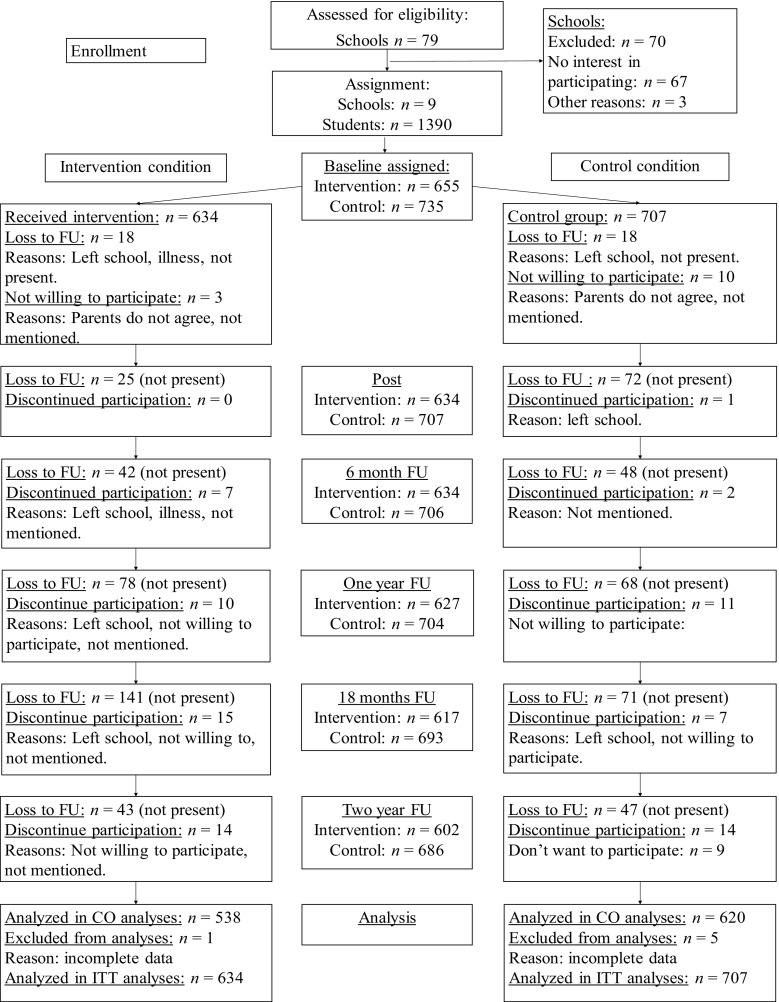
Participant flow

The sample consisted of 1390 students, see the Flow diagram for participant flow through the study (Fig. 1). Four schools (655 adolescents) were allocated to the intervention condition, see Table 1 for demographic characteristics. Only 0.5 % (three out of 655) adolescents declined participation. The control condition consisted of five schools (735 adolescents) where only 1.4 % (10 out of 735) declined to participate. Retention rates were high across all assessments: 96.5, 89.4, 89.3, 83.7, 77.4, and 84.5 % of participants completed the questionnaire at each assessment respectively. Generally, these high participation rates underscored the universal character of the study.

**Table 1:** Demographic characteristics

	Intervention condition *n* = 634	Control condition *n* = 707	Total *N* = 1341	Significant difference I-C
	*M* (*SD*)	*M* (*SD*)	*M* (*SD*)	*p*
Gender (%)				0.601
Girls	47.5	47.1	47.3	
Boys	52.5	52.9	52.7	
Age	13.95 (0.53)	13.86 (0.56)	13.91 (0.55)	0.045
Ethnicity (%)				0.002
Dutch	79.0	86.8	83.1	
Other ethnicity	21.0	13.2	16.9	
Education (%)				0.015
PVSE (low)	11.4	3.1	7.0	
HGSE (middle)	48.4	53.6	51.2	
PUE (high)	40.2	43.3	41.8	
Depressive symptoms	7.38 (5.62)	7.71 (5.77)	7.55 (5.70)	0.430
Elevated depressive symptoms (%)	13.2	15.1	14.2	0.833

Logistic regression analyses were used to calculate differences between I-C

*PVSE* pre-vocational secondary education (Dutch translation is VMBO), *HGSE* higher general secondary education (HAVO), *PUE* and pre-university education (VWO)

Level of depressive symptoms and elevated levels of depressive symptoms yes/no (CDI > = 13) were evenly represented in both conditions at baseline (Table 1). Lower education, *OR* = 0.31, 95 % CI = 0.18–0.52, *p* = .000, age, *OR* = 1.25, 95 % CI = 1.01–1.54, *p* = .043, and ethnicity *OR* = 0.67, 95 % CI = 0.50–0.91, *p* = .009, were unevenly distributed across condition and therefore we controlled for education, age, and ethnicity in subsequent analyses. Compared to the overall population in the Netherlands, the sample comprised fewer participants enrolled in pre-vocational secondary education (45.8 vs. 7.0 % of all students). This was due to the fact that only a few schools providing pre-vocational education agreed to participate in the study. The percentage of adolescents included from ethnic minorities was somewhat lower compared to the overall population, 16.9 % compared to 20.3 % respectively. Attrition analyses showed that adolescents were more likely to decline further participation in the study if they were female, *OR* = 0.50, 95 % CI = 0.30–0.83, *p* = .007, or if they were in the control condition, *OR* = 0.59, 95 % CI = 0.36–0.96, *p* = .033. Further, participants were less likely to drop out from the study when they were in PVSE compared to HGSE and PUE, *OR* = 0.19, 95 % CI = 0.07–0.51, *p* = .001, and when they were in HGSE compared to PVSE and PUE, *OR* = 0.19, 95 % CI = 0.01–0.38, *p* < .001.

Program fidelity was 80 %, which is fairly high, and comparable to other studies measuring fidelity through self-reports (Pössel et al. 2013). In a PRP prevention program fidelity was measured by coding audio tapes of PRP lessons, and was about 80 % as well (Gillham et al. 2007). On average, adolescents were present during 14–15 of the 16 lessons, which is much higher than in most other studies on PRP (e.g., Chaplin et al. 2006; Gillham et al. 2007). Of the adolescents participating in the study, 67.8 % indicated that they were present at the booster session. On average, adolescents did not like the program, *M* = 1.58, *SD* = 0.69, Range = 1–4, they did not think the skills were useful, *M* = 1.96, *SD* = 0.85, Range = 1–4, and the program did not made them feel happy, *M* = 1.48, *SD* = 0.67, Range = 1–4. In contrast, they thought the group atmosphere was rather safe to share their personal experiences, *M* = 2.46, *SD* = 1.06, Range = 1–4. OVK group trainers reported that adolescents were motivated, *M* = 2.64, *SD* = 1.07, Range = 1–5, applied the skills in daily life, *M* = 2.93, *SD* = 0.83, Range = 1–5, and listened well to the group trainers, *M* = 3.21, *SD* = 1.25, Range = 1–5. Group trainers pointed out that the groups were too large, *M* = 3.81, SD = 1.34, Range = 1–5, but characterized the group atmosphere as safe, *M* = 3.09, SD = 1.28, Range = 1–5.

Based on the univariate analyses (Table 2), adolescents in the intervention condition showed somewhat more depressive symptoms immediately following intervention compared to the control group, *t*(*df*) = 1195) = 1.86, *p* = .063. Moreover, adolescents in the intervention condition were more likely to have elevated depressive symptoms at post intervention than those in the control condition, *χ*^2^ (1) = 7.03, *p* = .008. At all other follow-up assessments, the intervention and control condition did not differ on level of depressive symptoms and number of elevated depressed adolescents. The iatrogenic effect at post assessment disappeared when controlling for baseline depressive symptoms, gender, age, ethnicity, school type, and the clustering of participants in schools; condition did not predict the level of depressive symptoms at post assessment in the completers only (CO), *β* = .03, *SE* = 0.05, *p* = .283, and Intent-to-treat framework (ITT), *β* = 0.06, *SE* = 0.05, *p* = .270. Also, condition did not predict the number of adolescents reporting elevated levels of depressive symptoms at post assessment in both CO, *OR* = 1.56, 95 % CI = 0.861–2.914, *p* = .139, and ITT analyses, *OR* = 0.50, 95 % CI = 0.90–3.02, *p* = .102. In all subsequent analyses, baseline depressive symptoms, gender, age, ethnicity, school type, and the clustering of participants in schools were controlled.

At 1 year follow-up, in the CO analyses the treatment and control condition did not differ in the level of depressive symptoms (Table 3), condition: *β* = −0.02, *SE* = 0.03, *p* = .653, Cohen’s *d* = 0.06, 95 % CI = −0.06–0.17, nor in the number of participants with elevated depressive symptoms, condition: *OR* = 0.94, 95 % CI = 0.58–1.55, *p* = .819, NNT = 137. This pattern was the same in the ITT analyses – depressive symptoms, condition: *β* = −0.01, *SE* = 0.05, *p* = .829, Cohen’s *d* = 0.02, 95 % CI = −0.08–0.13, elevated depressive symptoms, *OR* = 1.00, 95 % CI = 0.60–1.65, *p* = 0.992, NNT = 188.

**Table 2:** Difference in intervention and control condition in depressive symptoms at post intervention and follow-up: CO analyses

	Post			6 months FU			1 year FU			18 months FU			2 years FU		
	I	C	I-C	I	C	I-C	I	C	I-C	I	C	I-C	I	C	I-C
	*M* (*SD*)	*M* (*SD*)	*p*	*M* (*SD*)	*M* (*SD*)	*p*	*M* (*SD*)	*M* (*SD*)	*p*	*M* (*SD*)	*M* (*SD*)	*p*	*M* (*SD*)	*M* (*SD*)	*p*
Depressive Symptoms	8.4 (7.0)	7.7 (6.1)	0.063	7.5 (6.4)	7.9 (6.2)	0.392	8.3 (8.1)	8.8 (8.3)	0.333	8.3 (7.9)	8.9 (8.3)	0.282	8.4 (7.6)	8.0 (7.3)	0.325
	%	%	*p*	%	%	*p*	%	%	*p*	%	%	*p*	%	%	*p*
Elevated DS	20.6	14.9	0.008	14.1	13.9	0.939	17.7	18.8	0.631	18.1	19.4	0.590	20.2	16.5	0.103

Differences between conditions were tested with an independent sample *T*-test and a Chi-square test. In these analyses, the control variables, age, ethnicity, gender, school level and baseline depressive symptoms were not included, and the clustering of data in schools was not taken into account

*I* intervention condition, *C* control condition, *DS* depressive symptoms

**Table 3:** Regression analysis testing the effectiveness of OVK in reducing depressive symptoms and elevated depressive symptoms at 1 year follow-up

		Continuous outcome depressive symptoms									Dichotomous outcome elevated depressive symptoms					
		ITT (*N* = 1341)				CO (*N* = 1158)					ITT (*N* = 1341)			CO (*N* = 1158)		
Step		β	*SE*	*p*	*R* ^*2*^	β	*SE*	*p*	*R* ^*2*^	Step	*OR*	95 % CI	*R* ^2^	*OR*	95 % CI	*R* ^2^
1	Age	0.01	0.03	0.778	0.131	−0.02	0.02	0.380	0.139	1	1.13	0.87–1.45	0.109	0.97	0.80–1.17	0.113
	Ethnicity	0.03	0.03	0.217		0.04	0.02	0.034			1.13	0.75–1.70		1.19	0.79–1.79	
	ME	0.04	0.04	0.377		0.02	0.04	0.658			1.32	0.94–1.87		1.21	0.82–1.79	
	LE	0.02	0.02	0.275		0.02	0.01	0.024			1.94	1.21–1.97		2.41	1.52–3.83	
	Gender	0.11	0.03	0.000		0.12	0.02	0.000			1.48	1.12–1.97		1.48	1.17–1.87	
	DSB	0.35	0.04	0.000		0.37	0.04	0.000			5.00	3.57–7.01		6.06	4.69–7.84	
2	Condition	−0.01	0.05	0.829	0.131	−0.02	0.03	0.653	0.140	2	1.00	0.60–1.65	0.110	0.94	0.58–1.55	0.113
3	C*G	−0.01	0.05	0.883	0.132	−0.01	0.05	0.874	0.140	3	0.76	0.46–1.25	0.112	0.70	0.48–1.04	0.115
	C*DSB	0.001	0.04	0.975		0.03	0.03	0.390			0.86	0.44–1.71		0.81	0.53–1.25	

Logistic regression analyses were used for the dichotomous outcome elevated depressive symptoms

*ME* middle education, *LE* lower education, *DSB* depressive symptoms at baseline, *G* gender, *C* condition

In both CO and ITT analyses, a main effect of baseline depressive symptoms and elevated depressive symptoms was found, indicating that higher levels of depressive symptoms or having elevated levels of depressive symptoms at baseline was related to higher levels of depressive symptoms and having elevated depressive symptom levels at 1 year follow-up respectively. A main effect of gender was found in CO and ITT analyses, indicating that boys compared to girls reported higher levels at 1 year FU and showed a larger increase in depressive symptoms; the same pattern was also observed for elevated levels of depressive symptoms. In CO and ITT analyses it was found that adolescents in pre-vocational secondary education (PVSE) were more likely to report elevated depressive symptoms compared to adolescents in pre-university education (PUE). In CO analyses for level of depressive symptoms only, it was found that receiving PVSE compared to PUE, and having a non-native ethnic background were associated with more depressive symptoms. Neither baseline depressive symptoms nor gender moderated OVK’s effectiveness.

### Latent Growth Curve Modeling Testing the Longitudinal Effectiveness of OVK

Latent Growth curve modeling was conducted in the ITT framework. Since there was no variation on the mean of the quadratic slope, *M* = −0.001, *p* = .700, and the variance of the quadratic slope was very small, σ^2^ = 0.001, *p* = .001, only the linear slope was included in the models. The model showed a reasonable fit across the 20 datasets, RMSEA: *M* = 0.059, *SD* = 0.005; CFI: *M* = 0.892, *SD* = 0.016; SRMR: *M* = 0.045, *SD* = 0.004. At post assessment, the mean level of depressive symptoms was: intercept *M* = 0.30, *p* = .000. Depressive symptoms did not change across the follow-up assessments, LS: *M* = 0.01, *p* = .341. The variance of the intercept and linear slope were both significant, respectively: σ^2^ = 0.03, *p* = .000; σ^2^ = 0.002, *p* = .000, although small for the linear slope. This indicates that there were differences between participants on the value at post assessment (intercept) and also some differences in the rate of change across the follow-up period (LS).

In our second model, the control and predictor variables were added. This model showed a good fit across the 20 imputed datasets, RMSEA: *M* = 0.049, *SD* = 0.004; CFI: *M* = 0.903, *SD* = 0.013; SRMR: *M* = 0.026, *SD* = 0.001. Condition was not associated with the intercept or slope of depressive symptoms (Table 4), which means that adolescents in the control and intervention group did not differ in level of depressive symptoms at post intervention nor in the change across the follow-up assessments. Age, educational level, and baseline depressive symptoms were related to depressive symptoms at post intervention and to change across the follow-up assessments. Older adolescents and adolescents with higher levels of depressive symptoms at baseline showed higher levels of depressive symptoms at post intervention. Being older and reporting higher levels of depressive symptoms at baseline were related to a decrease in depressive symptoms across the follow-up. Adolescents in higher general secondary education (HGSE) reported fewer depressive symptoms at post intervention, but showed an increase in depressive symptoms over time compared to adolescents in PUE.

**Table 4:** Estimates of predictors and control variables for the intercepts and slopes of depressive symptoms across 2 year follow-up; intent-to-treat analyses

	Depressive symptoms	
	Intercept β (*p*-value)	Linear slope β (*p*-value)
Model 2		
Age	0.08 (0.004)	−0.12 (0.000)
Ethnicity	0.01 (0.587)	0.02 (0.787)
Middle education	−0.07 (0.047)	0.16 (0.022)
Lower education	0.02 (0.759)	0.10 (0.548)
Gender	0.07 (0.077)	0.04 (0.619)
DS at B	0.75 (0.000)	−0.22 (0.000)
Condition	0.02 (0.642)	−0.01 (0.919)
Model 3		
Condition * gender	0.08 (0.137)	−0.05 (0.728)
Condition * DS at B	−0.01 (0.841)	0.00 (0.970)

Gender: girls = 0, boys = 1. DS = depressive symptoms. B = baseline

Our third model tested whether gender and baseline depressive symptoms moderated intervention effects. This model showed a good fit, RMSEA: *M* = 0.049, *SD* = 0.004; CFI: *M* = 0.90, *SD* = 0.013; SRMR: *M* = 0.025, *SD* = 0.001. As presented in Table 4, gender and baseline depressive symptoms did not moderate intervention effects on the intercept or slope of depressive symptoms.

### Ancillary Analyses: Treatment Fidelity and Program Characteristics as Predictors of OVK Effectiveness

It was examined whether, within the intervention group, treatment fidelity and OVK program characteristics were associated with depressive symptoms at 1 year follow-up. In these analyses we controlled for baseline depressive symptoms and the clustering of participants in schools. None of the following variables were associated with prevention outcomes at 1 year follow-up: OVK group, *β* = −0.001, *SE* = 0.08, *p* = .990, group trainer, *β* = −0.01, *SE* = 0.03, *p* = .737, and number of lessons attended, *β* = −0.06, *SE* = 0.07, *p* = .400.

However, the degree to which adolescents felt the group atmosphere was safe to share their personal experiences was associated with lower levels of depressive symptoms at 1 year follow-up, *β* = −0.10, *SE* = 0.02, *p* = .000. This finding was also significant when the Bonferroni correction for multiple testing was performed *p* < .05/11 = 0.0045. This variable explained 5.6 % additional variance in depressive symptoms at 1 year follow-up in addition to the 12.4 % of variance explained by baseline depressive symptoms. None of the other adolescent feedback variables and none of the group trainer feedback variables explained any of the variance in level of depressive symptoms at 1 year follow-up.

## Discussion

In the present study, the longitudinal effectiveness of OVK as a universal school-based depression prevention program for adolescents was investigated. At 1 year follow-up and across the 2 year follow-up period, neither the level of depressive symptoms nor the number of adolescents reporting elevated levels of depressive symptoms differed across conditions. Gender and depressive symptom level at baseline did not moderate intervention effects, which implies that for both boys and girls, and for adolescents displaying high or low levels of depressive symptoms at baseline, OVK did not prevent depressive symptoms over the 2 year follow-up period. Univariate tests indicated that adolescents in the intervention group reported higher levels of depressive symptoms and were more likely to experience elevated levels of depressive symptoms. However, these differences at post intervention did not appear when randomization differences and the clustering of participants in schools were taken into account.

In contrast to other studies on PRP OVK was not effective in preventing depressive symptoms in early adolescence. This finding might be explained by differences in the samples studied. In previous studies demonstrating the effectiveness of PRP as a universal prevention program, less than 25 % of the originally targeted population participated (e.g., Chaplin et al. 2006; Gillham et al. 2006; Gillham et al. 2007). As the accepted definition of “universal prevention” is when all adolescents from a population are included (Merry et al. 2012), this raises the question as to what extent these PRP trials can be considered universal. Similarly, past PRP studies may have introduced a selection bias as adolescents in most studies actively chose to participate in PRP after school hours. In contrast, in the current study adolescents participated in OVK during school hours through passive consent, but were free to withdraw from the study. Although the mean level of depressive symptoms of the adolescents included in the PRP trial by Gillham et al. (2007) were comparable to symptom levels in community samples (Kovacs 2001) and to the current study, the participants in Gillham et al. (2007) may have differed from the current study on motivation to learn new skills, motivation to change behavior, hopefulness, support from parents, and experienced burden. Because of these differences, the participants in PRP may have benefited more from the program compared to adolescents in the current trial, even though the attendance rates were higher in OVK compared to PRP. These differences between the research samples potentially explain why past studies on PRP rendered positive outcomes whereas the current study did not.

Differences related to how depressive symptoms were measured might also explain why the current study did not render an effect. Universal studies (where 70 % or more of the population was included) that reported effects of CBT-based prevention did not use the CDI, but used the CES-D and SBB-DES (Pössel et al. 2004, 2008), or the RADS and the BDI-II (Merry et al. 2004). Some universal programs led to decreases in depressive symptoms when measured with the CDI, but these were studies which included less than 20 % of the targeted population (Chaplin et al. 2006; Gillham et al. 2007), were not randomized (Challen et al. 2014), or found effects for high risk groups only (Cutuli et al. 2006; Horowitz et al. 2007; Lowry-Webster et al. 2001; Sheffield et al. 2006; Stallard et al. 2012). Aspects related to the psychometric properties of the CDI might partly explain these results. Recently, the CDI was found to be less sensitive and reliable for detecting depressive symptoms in adolescents with low to mild levels of depressive symptoms compared to in adolescents with clinical levels of depressive symptoms (Lee et al. 2012). Since the majority of adolescents in universal prevention have low to mild depressive symptoms, the CDI may not have been sensitive enough to detect changes in depressive symptoms in our study.

Aspect of OVK’s program content could have also resulted in less favorable prevention outcomes. For instance, some aspects of OVK’s program could be improved to make the program more appealing to youth. First, the program might be too long; adolescents might lose interest and motivation over the course of the program, and it has been found that longer prevention programs have fewer positive outcomes (Stice et al. 2009). Second, the exercises in OVK were often described as relatively boring by the students; adolescents would have liked more active role playing exercises and to perform more exercises electronically. Third, students pointed out that they would like to discuss more examples from their own lives instead of the examples provided in the program. By discussing their own issues, the skills taught by OVK might become more relevant and may more easily transfer to adolescents’ daily lives. Moreover, sharing emotional information could enhance group cohesion and perceived group safety. Past research indicates that group cohesion is positively associated with treatment outcomes (Crowe and Grenyer 2008). In the current study, adolescents who felt safe to share their experiences reported fewer depressive symptoms at 1 year follow-up.

Contrary to expectations and previous research on PRP, adolescents who reported elevated depressive symptoms at baseline did not benefit more from OVK compared to other adolescents. A factor that might explain these results could be the group climate. In selective or indicated prevention, the group climate and content is different compared to in universal contexts. In targeted programs, people share their stories and feel strengthened by others because they realize that they are not the only one experiencing those feelings. Sharing emotions increases the group’s intimacy which fosters group cohesion (Yalom and Leszcz 2008). In universal prevention groups, however, adolescents with high levels of depressive symptoms might realize they are different from the rest, and may therefore not share their experiences. Supporting this idea, when implemented as an indicated prevention program, OVK was highly effective in reducing depressive symptoms in adolescent girls who had subclinical or clinical symptom levels at baseline (Wijnhoven et al. 2014). In this study, female students (13–14 years) received the first eight lessons of OVK in all-girl groups that were held at school but outside school hours. In sum, it seems important to tailor the program to the needs of the adolescents; the group composition, the number of lessons, the topics addressed, and the context in which the program is provided should fit the needs of the participating adolescents in order to effectively prevent depressive symptoms.

### Strengths & Limitations

The main strength of the current study is the randomized controlled design to test program effectiveness (Schulz et al. 2010). Second, this is the first universal randomized controlled study to test longitudinal outcomes of a prevention program based on PRP. Third we included a large sample. Fourth, we were able to maintain response rates between 77.4 and 96.5 % at all six assessments. Finally, the theoretical basis of the program is strong, since it is based upon evidence-based practices that can effectively reduce or prevent depressive symptoms (Calvete et al. 2011; Klein et al. 2007).

The fact that treatment fidelity was assessed by solely relying on self-reports can be considered a limitation of the study, as group trainers may have the tendency to answer in a socially desirable way. Preferably, program fidelity should be measured by tape-recording lessons and scoring those lessons by independent coders. Still, group trainers were frequently reminded of the importance of following the protocol and honestly reporting program fidelity. Another limitation of the study was the inclusion of schools as the unit of randomization instead of individuals, since this increased the risk of participant characteristics differing across the intervention and control groups. To take this into account, we checked for randomization differences and controlled for any differences in our analyses. On the other hand, using schools as the unit of randomization minimizes contamination between research conditions, since all adolescents included in a certain school are in the same condition. A third limitation of this study was the use of only one self-report measure of depressive symptoms. Although self-reports are a valid, reliable, and widely used method to study depressive symptoms (Kovacs 2001), reports from parents, teachers, and clinicians could have provided a more comprehensive picture. Since the CDI might be less sensitive and reliable for detecting low to mild depressive symptoms, a different self-report measure could have been used. Other limitations concern the generalizability of this study given that relatively few participants followed pre-vocational secondary education (PVSE) in contrast to higher educational levels, and relatively few adolescents with a different ethnic background participated in the study. Generalizing our findings to the normal population should therefore be done with caution.

### Implication & Future Research

This study is not the first to report null effects for a universal prevention program designed to prevent depressive symptoms (e.g., Sawyer et al. 2010). Although some studies indicated short term effects (e.g., Spence et al. 2005), or even long term effects (Lock and Barrett 2003; Merry et al. 2004; Pössel et al. 2004, 2008, 2013; Shochet et al. 2001), the impact of these effects on the lives of adolescents was often small. Therefore, other approaches for preventing depression should be considered. For instance, a stepped care approach could be promising, which incorporate a screening for depressive symptoms for all adolescents, and targeted or indicated prevention provided to the ones at need (Silverstone et al. 2015). Including a screening has several advantages, the ones experiencing elevated levels of depressive symptoms are identified, it could increase awareness of internalizing problems and hope for change, and it might creating a culture in which internalizing problems can be talked about. Adolescents indeed show a decrease in depressive symptoms following screening (Wijnhoven et al. 2014). However, in addition to effectiveness studies, cost-effectiveness analyses should be conducted to reveal whether it is worthwhile to implement a stepped care approach including a broad screening. With respect to the mode of delivery of prevention programs, game-based interventions could be included, since games have the potential to engage adolescents which could enable learning and increase prevention effects (Granic et al. 2014). To further improve effective selective and indicated depression prevention programs, future research should study the moderators and mediators of these programs.

## Conclusion

When implemented as a universal, school-based prevention program, OVK was not effective at preventing depressive symptoms over the course of the 2 year follow-up. Based on these and other previous studies, implementing universal depression prevention programs and conducting new trials testing universal depression prevention programs is not recommended.
